# Overexpression of miR-29a-3p Suppresses Proliferation, Migration, and Invasion of Vascular Smooth Muscle Cells in Atherosclerosis via Targeting TNFRSF1A

**DOI:** 10.1155/2020/9627974

**Published:** 2020-09-04

**Authors:** Liyi You, Hao Chen, Lixin Xu, Xun Li

**Affiliations:** ^1^Department of Cardiology, The First Affiliated Hospital of Soochow University, Suzhou, 215006 Jiangsu, China; ^2^Department of Cardiology, Wenzhou People's Hospital, The Wenzhou Third Clinical Institute Affiliated to Wenzhou Medical University, Wenzhou, 325000 Zhejiang, China; ^3^Department of Ultrasound, Wenzhou People's Hospital, The Wenzhou Third Clinical Institute Affiliated to Wenzhou Medical University, Wenzhou, 325000 Zhejiang, China

## Abstract

**Objective:**

Increasing evidence highlights the significance of microRNAs (miRNAs) in the progression of atherosclerosis (AS). Our aim was to probe out the role and regulatory mechanism of miR-29a-3p in AS.

**Methods:**

An in vivo model of AS was conducted by high-fat diet ApoE^−/−^ mice. Oxidized low-density lipoprotein- (ox-LDL-) exposed vascular smooth muscle cells (VSMCs) were utilized as an in vitro of AS. Serum levels of total cholesterol (TC), triglyceride (TG), low-density lipoprotein cholesterol (LDL-C), and high-density lipoprotein cholesterol (HDL-C) were detected. Hematoxylin and eosin (H&E) and Masson's staining was presented to investigate the pathological changes. miR-29a-3p and TNFRSF1A expression was detected by RT-qPCR. Proliferative, migrated, and invaded abilities of VSMCs were determined via a series of assays. The interaction between miR-29a-3p and TNFRSF1A was verified through luciferase reporter assay.

**Results:**

Upregulated miR-29a-3p and downregulated TNFRSF1A were found both in vitro and in vivo models of AS. miR-29a-3p mimic distinctly decreased the serum levels of TC, TG, and LDL-C and increased serum HDL-C levels. Moreover, its overexpression could ameliorate plaque formation of AS mice. In ox-LDL-induced VSMCs, miR-29a-3p overexpression notably decreased cell proliferation, migration, and invasion, which was reversed by TNFRSF1A overexpression. Also, miR-29a-3p could directly target the 3′UTR of TNFRSF1A.

**Conclusion:**

miR-29a-3p overexpression ameliorated plaque formation of AS and suppressed proliferation, migration, and invasion of ox-LDL-induced VSMCs via TNFRSF1A, which offered novel insights into the progression of AS.

## 1. Introduction

Atherosclerosis (AS) is a chronic vascular inflammatory disease related with endothelial dysfunction [1]. Despite the progress made in lifestyle management and medication, total mortality of AS exceeds 50% in developed countries. Thus, there is an urgent need to clarify the molecular mechanisms of AS and to determine effective treatment options. AS is triggered by various risk factors, including the aberrant proliferation of VSMCs. The pathological proliferation of VSMCs has been shown to accelerate AS progression and restenosis of arteries [2]. VSMC dysfunction plays a crucial function in the thickening of atherosclerotic intima. During AS, abnormal proliferation of VSMCs participates in the formation of atherosclerotic plaques [3]. Thus, it is essential to fully understand the behaviors of VSMCs in AS for determining therapeutic targets for the prevention and treatment of AS.

Ox-LDL is a common risk factor for AS, which accelerates the formation of atherosclerotic plaques as well as VSMC migration and proliferation. miRNAs are a class of highly conserved noncoding endogenous RNAs, approximately 22 nucleotides in length. They can bind 3′ untranslated region (3′UTR) of a specific-target mRNA sequence, thereby mediating posttranscriptional gene expression [4]. miRNAs may be crucial in affecting the function of VSMCs, driving atherosclerotic plaque formation and cholesterol homeostasis. Studies have confirmed that the abnormal expression patterns of several miRNAs promote the progression of AS, such as miR-144 [5], miR-155 [6], and miR-148b [7]. The function of miR-29a-3p in the pathogenesis of cardiovascular diseases (like myocardial fibrosis [8], cardiac ischemia-reperfusion [9], and cardiac hypertrophy [10]) has attracted wide attention. Nevertheless, the role of miR-29a-3p in AS remains unclear.

TNF receptor-1 (TNFRSF1A) has been confirmed to be in association with the progression of various cardiac diseases, including OS. It can mediate endothelial cell dysfunction and inflammation [11]. TNFRSF1A could accelerate the exacerbation of AS [12]. Clinical trial results demonstrated that high circulating TNFR2 level in patients with stable coronary heart disease is in association with increased risk of cardiovascular events and death [13]. Nevertheless, the regulatory mechanism of TNFRSF1A is not well uncovered. In this study, we investigated the role of miR-29a-3p and TNFRSF1A in vitro and in vivo models of AS. Furthermore, the interactions between miR-29a-3p and TNFRSF1A were identified for AS in this study.

## 2. Materials and Methods

### 2.1. Animal Models

A total of twenty 8-week-old ApoE^−/−^ male mice (SCXK (Su) 2018-0008) were purchased from Jiangsu Jicui Yaokang Biotechnology Co., Ltd. (Jiangsu, China). High-fat feed was composed of 78% basic feed, 10% egg yolk powder, 2% cholesterol, and 10% lard (TP28522; Nantong Trophy Feed Technology Co., Ltd., Jiangsu, China). All mice were randomly allocated into four groups. Three groups of mice were fed free high-fat diet for 12 weeks. At the same time, the remaining group of mice was fed basic diet normally. All mice were fed freely and kept in an environment with a day-night cycle of 12 hours, a temperature of 22-25°C, and a humidity of 50-70%. At the 10th week of modeling, 15 mice freely fed high-fat diet were randomly divided into 3 groups (AS group, AS+NC mimic group, and AS+miR-29a-3p mimic group). Briefly, mice were injected with 50 *μ*g adenovirus vector NC mimic and miR-29a-3p mimic (General Biotechnology Co., Ltd., Anhui, China) through the jugular vein every other day for 12 weeks and continued to be fed a high-fat diet. By week 12, all mice were euthanized. Peripheral blood was collected, and smooth muscle cells were isolated. Our research was approved by the Animal Ethics Committee of The First Affiliated Hospital of Soochow University (2019033).

### 2.2. RT-qPCR Assay

1 − 5 × 10^7^ cells were lysed by 1 ml TRIzol (R1100, Solarbio, Beijing, China) at room temperature for 5 min. After centrifugation, the precipitate was treated by 0.2 ml chloroform for 2 min. After centrifugation at 12,000g for 15 min at 4°C, the supernatant was harvested. The concentration of RNA extract was then determined according to the OD260/OD280 ratio. RNA was reverse transcribed into cDNA using the reverse transcription kit (11123ES60, YEASEN, Shanghai, China). qPCR was presented using the qPCR kit (11201ES03, YEASEN). The primer sequences were as follows: mmu-miR-29a-3p, 5′-CGTAGCACCATCTGAAATCG-3′ (forward), 5′-GTGCAGGGTCCGAGGT-3′ (reverse); mmu-U6, 5′-CGCAAGGATGACACGCAAAT-3′ (forward), 5′-GCAGGGTCCGAGGTATTC-3′ (reverse); human-miR-29a-3p, 5′-AGCACCAUCUGAAAUCGGUUA-3′ (forward), 5′-GTGCAGGGTCCGAGGT-3′ (reverse); human-U6, 5′-CTCGCTTCGGCAGCACA-3′ (forward), 5′-AACGCTTCACGAATTTGCGT-3′ (reverse); mmu-TNFRSF1A, 5′-GGTCTTTGCCTTCTATCCTTTATC-3′ (forward), 5′-CTTTCCAGCCTTCTCCTCTTTG-3′ (reverse); human-TNFRSF1A, 5′-GAGAATGTTAAGGGCACTGAG-3′ (forward), 5′-CCCACAAACAATGGAGTAGA-3′ (reverse); mmu-actin, 5′-ATGTGCGACGAAGACGAGAC-3′ (forward), 5′-CCTTCTGACCCATACCTACCAT-3′ (reverse); and human-actin, 5′-AATCGTGCGTGACATTAAGGAG-3′ (forward), 5′-ACGTGTTGGCGTAACAGGTCTT-3′ (reverse). The relative expression was calculated with the 2^–*ΔΔ*Ct^ method.

### 2.3. Hematoxylin and Eosin (H&E) and Masson's Staining

Fresh tissue was fixed in 4% paraformaldehyde for over 24 h. After dehydration and paraffin embedding, the tissue was cut to a thickness of 4 *μ*m. After paraffin sections were dewaxed, H&E staining was presented. The sections were stained with hematoxylin (G1004, Servicebio, Wuhan, China) for 5 min and 0.5% eosin solution (G1001, Servicebio) for 2 min. For Masson's staining, the sections were soaked in iron hematoxylin staining solution (G1006-2/3, Servicebio) for 3 min and Lichun acid magenta staining solution (G1006-4, Servicebio) for 5 min. After rinsing with tap water, the sections were stained with phosphomolybdic acid (G1006-5, Servicebio) for 3 min, aniline blue (G1006-6, Servicebio) for 5 min, and differentiated with 1% glacial acetic acid for 1 min. Following dehydration, the sections were mounted with neutral gum (10004160, Sinopharm, Shanghai, China) and placed under an optimal microscope (Nikon Eclipse CI, Nikon, Japan) for observation.

### 2.4. Biochemical Test

Serum levels of total cholesterol (TC), triglyceride (TG), low-density lipoprotein cholesterol (LDL-C), and high-density lipoprotein cholesterol (HDL-C) were determined using mouse the TG kit (AUTEC), mouse LDL-C kit (LABO, Germany), and HDL-C kit (LABO, Germany), respectively.

### 2.5. Cell Culture and Treatment

Human VSMCs from umbilical artery from American-type culture collection (ATCC; Rockville, Maryland) were cultured in DMEM (11965092, Gibco, New York, USA) plus 10% fetal bovine serum (10270-106, Gibco) at 37°C with 5% CO_2_. VSMCs were treated with different concentrations of ox-LDL (0, 25 *μ*g/ml, 50 *μ*g/ml, and 100 *μ*g/ml). miR-29a-3p mimic, pcDNA3.1-TNFRSF1A, and their controls were purchased from the GenePharma (Shanghai, China). Cells were transfected with 30 nM oligonucleotides via Lipofectamine 2000 (11668019, Thermo, Shanghai, China).

### 2.6. Cell Counting Kit-8 (CCK-8)

Cells were seeded onto a 96-well plate (TCP011096, Guangzhou, China), at the density of 5 × 10^3^ cells/well for 24 h. After discarding the cell culture medium, 100 *μ*l fresh medium containing 0.5% FBS as well as and 10 *μ*l CCK-8 kit (C0038, Beyotime, Beijing, China) was added to each well to each well and incubated at 37°C for 2 h. Optical density value was determined using a microplate reader (RT-6000, KAYTO, USA) at the 450 nm.

### 2.7. Western Blot

Tissues or cells were lysed with 200 *μ*l of RIPA lysate (BR0002, BEST, Xian, China) plus 1 mM PMSF at 4°C for 30 min. After centrifugation at 12,000 rpm for 10 min at 4°C, the supernatant was harvested and stored at -80°C. The BCA method was used to determine protein concentration using the BCA quantitative kit (P0012, Beyotime, Shanghai, China). Protein samples were subjected to polyacrylamide gel electrophoresis, which were then transferred onto PVDF membrane (IPVH00010, Millipore, Massachusetts, USA). The membrane was blocked by 5% skimmed milk powder at room temperature for 2 h. Then, the membrane was incubated with primary antibodies including Ki67 (ab92742, Abcam), proliferating cell nuclear antigen (PCNA; 10205-2-AP, ProteinTech, Chicago, USA), *β*-actin (ab8227, Abcam, Cambridge, United Kingdom), matrix metalloproteinase- (MMP-) 2 (10373-2-AP, ProteinTech), MMP9 (ab38898, Abcam), and TNFRSF1A (ab90463, Abcam) at 4°C overnight, followed by goat anti-rabbit IgG-HRP (BK0027, BEST) and goat anti-mouse anti-rabbit IgG-HRP (BK0023, BEST) secondary antibodies at room temperature for 1.5 h. The blots were visualized using the ECL Plus Luminous Kit (S17851, Yeasen, Shanghai, China).

### 2.8. Wound Healing Assay

Cells were seeded onto a 6-well plate. When the cells were fused 60%-80%, 1% double antibody serum-free medium was replaced. After 12-18 h, a straight line was scratched in the center of each well. After washing twice with phosphate-buffered solution (PBS), DMEM was replaced. The plate was cultured in a 5% CO_2_ incubator at 37°C. At 0 h and 48 h, the images were observed and taken.

### 2.9. Transwell Assay

Cell migration and invasion were determined using the Transwell assay. 600 *μ*l 12% FBS was added to the 24-well plate, followed by Transwell chamber (3422, Thermo, Shanghai, China). Then, 600 *μ*l FBS was added to the lower layer of Transwell chamber, and 4 × 10^4^ cells were added to the upper layer. For cell invasion assay, 100 *μ*g/ml Matrigel (356234, Franklin Lake, New Jersey, USA) was added to the upper layer of chamber. After 24 h of cultivation, the cells in the upper chamber were removed. The sample was fixed with 600 *μ*l fixative solution for 10 min. The chamber was stained with 600 *μ*l crystal violet (G1014, Servicebio, Wuhan, China) for 15 min. Finally, the results were photographed and counted under an inverted microscope (IX51, Olympus, Japan).

### 2.10. Dual-Luciferase Report

The construction of TNFRSF1A's wild-type (WT) and mutation-type (Mut) vectors was completed by General Bio. The experimental groups were as follows: TNFRSF1A-3′UTR-WT + NC mimic; TNFRSF1A-3′UTR-WT + miR-29a-3p mimic; TNFRSF1A-3′UTR-Mut and NC mimic; and TNFRSF1A-3′UTR-WT + miR-29a-3p mimic. Four to six hours before transfection, the complete medium was replaced with serum-free basic medium, and the cells were starved. 0.8 *μ*g plasmid, 2 *μ*l Lipofectamine 2000 (11668019, Thermo, Shanghai, China), and 20 pmol of miR-29a-3p mimic were cocultured with 50 *μ*l OPTI-MEM (31985088, Gibco, California, United States) for 5 min, respectively. The above solution was mixed and cultured at room temperature for 20 min. Following the removal of the cell culture medium, 450 *μ*l of fresh medium was added to the cells. After 48 h of culture, the cells were lysed by100 *μ*l passive lysis buffer at room temperature for 20 min. In line with the instructions of the Promega dual fluorescence reporter (E1910, Promega, Madison, Wisconsin, USA), the total amount of LAR II and Stop & GLo Reagent reagents was calculated. 10 *μ*l cell lysate was added into the microtiter plate, followed by 50 *μ*l LAR II. The firefly luciferase value was read using GLoMax 20/20 detector (Promega). 50 *μ*l Stop & GLo Reagent was then added, and the Renilla luciferase value was determined. The ratio firefly/Renilla luciferase value was calculated as the dual-luciferase activity.

## 3. Results

### 3.1. Overexpression of miR-29a-3p Distinctly Reduces Aorta Plaque Formation for AS Mice

**Figure 1:**
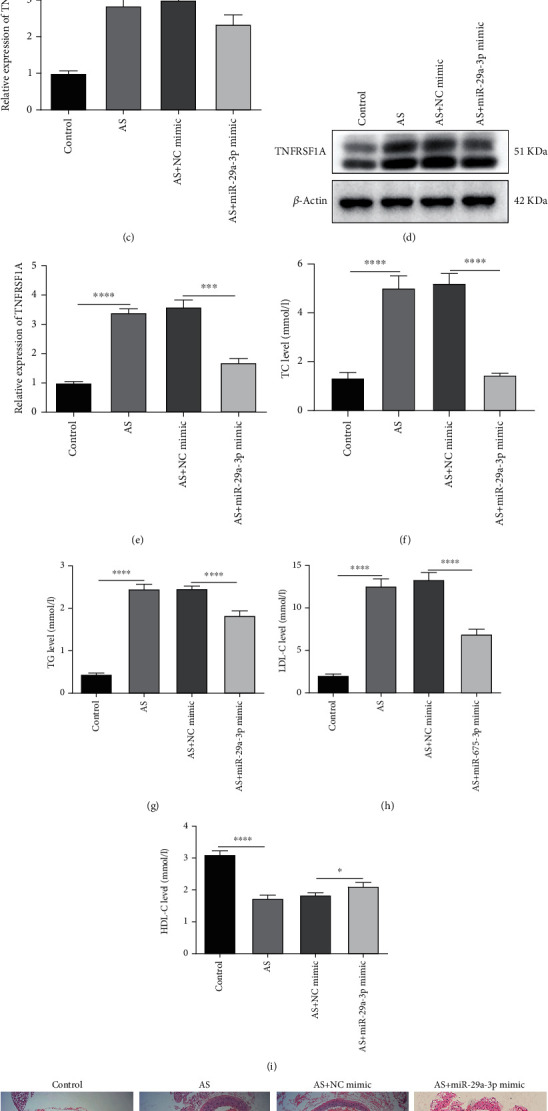
Overexpression of miR-29a-3p distinctly reduces aorta plaque formation for atherosclerosis (AS) mice. (a) Serum miR-29a-3p levels were examined in AS mice with or without miR-29a-3p mimic injection using RT-qPCR. (b, c) RT-qPCR was presented to detect miR-29a-3p as well as TNFRSF1A expression in AS mice with or without miR-29a-3p mimic injection. (d, e) TNFRSF1A protein expression was tested in AS mice with or without miR-29a-3p overexpression via western blot. (f–i) Biochemical test was performed to test the serum levels of total cholesterol (TC), triglyceride (TG), low-density lipoprotein cholesterol (LDL-C), and high-density lipoprotein cholesterol (HDL-C) in AS mice with or without miR-29a-3p mimic injection. (j) Representative images of H&E staining for aorta of AS mice. (k) Representative images of Masson's staining results for aorta of AS mice. ∗*p* < 0.05; ∗∗∗*p* < 0.001; ∗∗∗∗*p* < 0.0001.

In this study, we conducted an AS model using ApoE^−/−^ male mice. In Figures 1(a) and 1(b), miR-29a-3p expression was distinctly reduced in the serum and aorta vascular tissues of the AS model. As expected, miR-29a-3p mimic notably increased its expression in the AS model. We also found that there was an increased mRNA expression level of TNFRSF1A in the AS model compared to controls (Figure 1(c)). However, for AS mice injected with miR-29a-3p mimic, TNFRSF1A mRNA expression was significantly reduced (Figure 1(c)). Similarly, TNFRSF1A protein expression was significantly elevated in the AS model, which was reversed by miR-29a-3p mimic (Figures 1(d) and 1(e)). Biochemical test results demonstrated that there were higher serum levels of TC (Figure 1(f)), TG (Figure 1(g)), and LDL-C (Figure 1(h)) and lower HDL-C (Figure 1(i)) in the AS model compared to controls. However, after treatment with miR-29a-3p mimic, the serum levels of TC (Figure 1(f)), TG (Figure 1(g)), LDL-C (Figure 1(h)), and HDL-C (Figure 1(i)) were prominently reversed in the AS model. As shown in H&E results, the aorta vascular wall was smooth, and there was no atheromatous plaque in the vascular intima for mice in the control group (Figure 1(j)). In converse, there were obvious plaques in the AS mice. For AS mice treated with miR-29a-3p mimic, plaques were distinctly suppressed compared to those with NC mimic. Masson's staining results demonstrated that there was no atheromatous plaque formation in the vascular intima of the control mice (Figure 1(k)). In the AS model, the intima of the arteries was significantly thickened, and there were fibrous and lipid plaques. Furthermore, the surface of the plaque was covered with collagen fibers, and there were thin fiber cap and few collagen fibers for AS mice. Intriguingly, miR-29a-3p mimic injection prominently reduced plaque formation of AS mice.

### 3.2. Overexpression miR-29a-3p Suppresses Proliferation of ox-LDL-Induced VSMCs in AS

**Figure 2:**
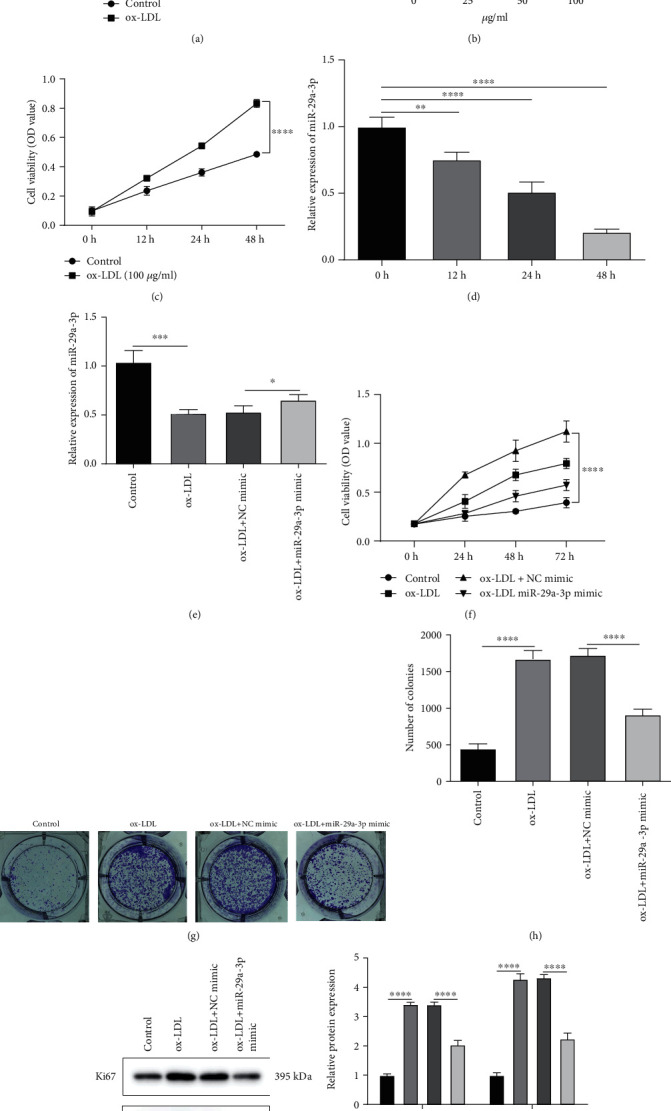
Overexpression miR-29a-3p suppresses proliferation of ox-LDL-induced vascular smooth muscle cells (VSMCs) in AS. (a) CCK-8 assay was used to detect the cell viability of VSMCs under different concentrations of ox-LDL. (b) miR-29a-3p expression was determined in VSMCs induced by different concentrations of ox-LDL by RT-qPCR. (c) The cell viability of VSMCs induced by 100 *μ*g/ml ox-LDL was detected using CCK-8 assay in different time periods. (d) RT-qPCR was presented to examine the expression of miR-29a-3p in VSMCs induced by100 *μ*g/ml ox-LDL in different time periods. (e) miR-29a-3p expression was tested in ox-LDL-induced VSMCs with or without miR-29a-3p mimic transfection by RT-qPCR. (f) CCK-8 assay was presented in ox-LDL-induced VSMCs with or without miR-29a-3p mimic transfection. (g, h) Colony formation assay was used to examine the proliferative ability of ox-LDL-induced VSMCs after transfection with miR-29a-3p mimic or not. (i, j) The expression of Ki67 and PCNA proteins was examined in ox-LDL-induced VSMCs with or without miR-29a-3p mimic transfection. ∗*p* < 0.05; ∗∗*p* < 0.01; ∗∗∗*p* < 0.001; ∗∗∗∗*p* < 0.0001.

VSMCs were exposed by different concentrations of ox-LDL to induce AS. CCK-8 was utilized the appropriate concentration of ox-LDL. In Figure 2(a), cell viability of VSMCs was notably increased with a concentration manner. Moreover, the expression of miR-29a-3p was significantly reduced with a concentration-dependent manner (Figure 2(b)). When the concentration of ox-LDL was 100 *μ*g/ml, VSMCs had the highest cell viability, and miR-29a-3p expression had the lowest expression level. Thus, 100 *μ*g/ml ox-LDL was determined for further analysis. When induced by 100 *μ*g/ml ox-LDL, the cell viability of VSMCs was distinctly increased, with a time-dependent manner (Figure 2(c)). Also, as time went by, miR-29a-3p expression was gradually reduced (Figure 2(d)). In ox-LDL-induced VSMCs, its expression was elevated after transfection by miR-29a-3p mimic (Figure 2(e)). CCK-8 results suggested that miR-29a-3p mimic prominently suppressed the viability of VSMCs induced by ox-LDL (Figure 2(e)). Furthermore, colony formation assay results showed that the proliferative ability of VSMCs was induced by ox-LDL, which was reversed by miR-29a-3p mimic (Figures 2(g) and 2(h)). We also detected the expression of proliferation-related proteins including Ki67 and PCNA in VSMCs. As expected, the expression of Ki67 and PCNA was significantly increased in VSMCs exposed by ox-LDL, which was distinctly reduced by miR-29a-3p mimic (Figures 2(i) and 2(j)).

### 3.3. Overexpression of miR-29a-3p Reduces Migration as Well as Invasion of ox-LDL-Induced VSMCs in AS

**Figure 3:**
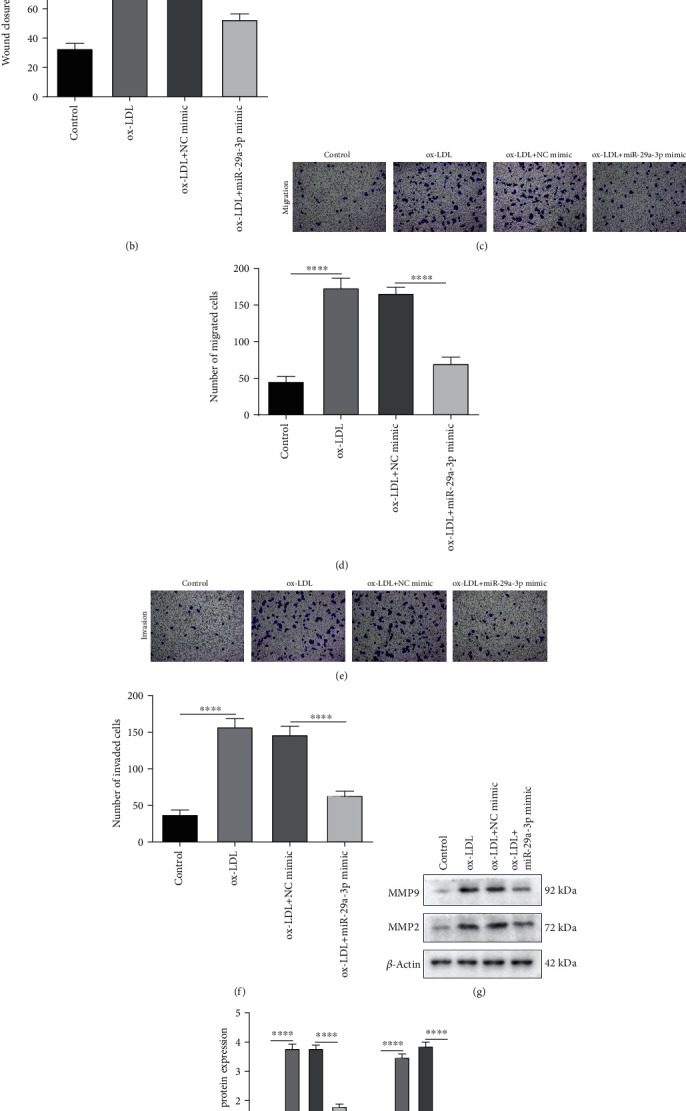
Overexpression of miR-29a-3p inhibits migration and invasion of ox-LDL-induced vascular smooth muscle cells (VSMCs) in AS. (a, b) Wound healing assay was utilized to examine the migrated ability of ox-LDL-induced VSMCs with or without miR-29a-3p mimic transfection. (c–f) The migrated and invaded abilities of VSMCs were determined using Transwell assays. (g, h) Western blot was presented to investigate the expression of MMP9 and MMP2 proteins in ox-LDL-induced VSMCs with or without miR-29a-3p mimic. ∗∗∗*p* < 0.001; ∗∗∗∗*p* < 0.0001.

Wound healing assay results demonstrated that ox-LDL-induced VSMCs had significantly higher migrated ability compared to control (Figures 3(a) and 3(b)). However, miR-29a-3p mimic distinctly suppressed the migrated abilities of VSMCs induced by ox-LDL (Figures 3(a) and 3(b)). As shown in Transwell assay results, ox-LDL exposure notably elevated the migrated and invaded abilities of VSMCs, which were reversed by miR-29a-3p overexpression (Figures 3(c)–3(f)). The expression of MMP9 and MMP2 proteins was examined in VSMCs using western blot. In Figures 3(g) and 3(h), their expression was elevated in VSMCs induced by ox-LDL, which was decreased by miR-29a-3p overexpression. The above findings suggested that miR-29a-3p could inhibit migration and invasion of ox-LDL-induced VSMCs in AS.

### 3.4. Overexpression of miR-29a-3p Suppresses Proliferation of ox-LDL-Induced VSMCs by Directly Targeting TNFRSF1A in AS

**Figure 4:**
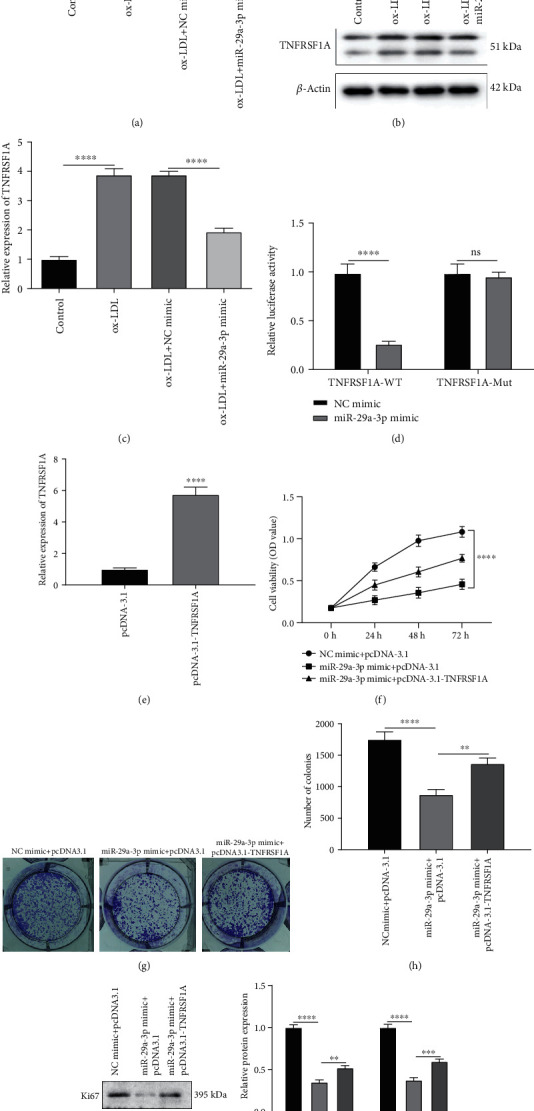
Overexpression of miR-29a-3p decreases proliferation of ox-LDL-induced vascular smooth muscle cells (VSMCs) by directly targeting TNFRSF1A in AS. (a–c) TNFRSF1A mRNA and protein expression was detected in ox-LDL-induced VSMCs with or without miR-29a-3p mimic injection using RT-qPCR and western blot. (d) Dual-luciferase report verified the direct interaction between miR-29a-3p and TNFRSF1A. (e) RT-qPCR was utilized to validate the transfection effect of pcDNA3.1-TNFRSF1A in ox-LDL-induced VSMCs. (f) CCK-8 assay was presented to examine the cell viability of ox-LDL-induced VSMCs injected with miR-29a-3p mimic and/or pcDNA3.1-TNFRSF1A. (g, h) Cell proliferation of ox-LDL-induced VSMCs was detected following transfection with miR-29a-3p mimic and/or pcDNA3.1-TNFRSF1A through colony formation assay. (i, j) Ki67 and PCNA expression in ox-LDL-induced VSMCs transfected with miR-29a-3p mimic and/or pcDNA3.1-TNFRSF1A. ∗∗*p* < 0.01; ∗∗∗*p* < 0.001; ∗∗∗∗*p* < 0.0001; ns: no statistical significance.

The mRNA and protein expression levels of TNFRSF1A were remarkably increased in ox-LDL-induced VSMCs compared to controls, which were decreased following miR-29a-3p overexpression (Figures 4(a)–4(c)). The dual-luciferase report confirmed that miR-29a-3p could target the 3′UTR of wide-type TNFRSF1A (Figure 4(d)). miR-29a-3p mimic conspicuously decreased the wide-type TNFRSF1A expression. To further probe out the interaction between miR-29a-3p and TNFRSF1A, TNFRSF1A was successfully overexpressed according to RT-qPCR (Figure 4(e)). CCK-8 results showed that TNFRSF1A overexpression could reverse the reduction of cell viability caused by miR-29a-3p overexpression in ox-LDL-induced VSMCs (Figure 4(f)). Furthermore, colony formation assay results showed that TNFRSF1A overexpression could remarkably changeover the decrease in proliferative abilities by miR-29a-3p overexpression in ox-LDL-induced VSMCs (Figures 4(g) and 4(h)). As expected, miR-29a-3p mimic notably decreased Ki67 and PCNA expression, which was reversed following TNFRSF1A overexpression in ox-LDL-induced VSMCs (Figures 4(i) and 4(j)).

### 3.5. Upregulation of miR-29a-3p Inhibits Migration as well as Invasion of ox-LDL-Induced VSMCs via Directly Targeting TNFRSF1A in AS

**Figure 5:**
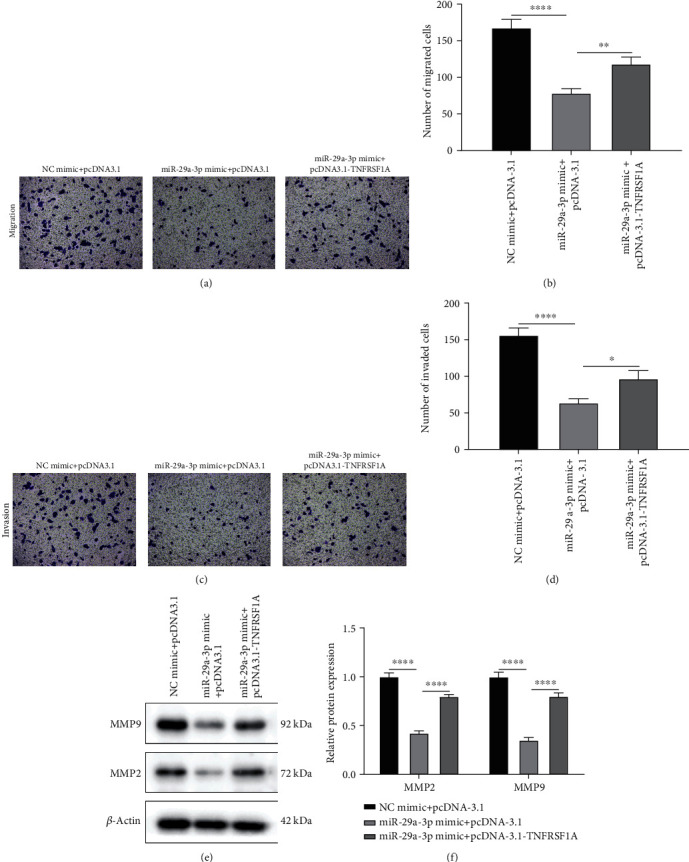
Upregulated miR-29a-3p inhibits migration as well as invasion of ox-LDL-induced vascular smooth muscle cells (VSMCs) via directly targeting TNFRSF1A in AS. (a–d) Transwell assays were carried out to examine the number of migrated and invaded ox-LDL-induced VSMCs in ox-LDL-induced VSMCs transfected with miR-29a-3p mimic and/or pcDNA3.1-TNFRSF1A. (e, f) MMP9 and MMP2 protein expression in ox-LDL-induced VSMCs. ∗*p* < 0.05; ∗∗*p* < 0.01; ∗∗∗∗*p* < 0.0001.

In Figures 5(a) and 5(b), miR-29a-3p overexpression obviously lessened the number of migrated ox-LDL-induced VSMCs, which was reversed following pcDNA3.1-TNFRSF1A. Moreover, TNFRSF1A overexpression could reverse the reduction of the number of invaded ox-LDL-induced VSMCs caused by miR-29a-3p overexpression (Figures 5(c) and 5(d)). The expression of MMP9 and MMP2 proteins was reduced in ox-LDL-induced VSMCs following miR-29a-3p overexpression, which was reversed following pcDNA3.1-TNFRSF1A (Figures 5(e) and 5(f)). These findings revealed that miR-29a-3p overexpression could cut down migration and invasion of ox-LDL-induced VSMCs via directly targeting TNFRSF1A in AS.

## 4. Discussion

In our study, ApoE^−/−^ mice was used to conduct the AS model using high-fat diet [14, 15]. miR-29a-3p was prominently lowly expressed in the AS mouse model. Increasing evidence suggests that abnormal expression miR-29a-3p is in association with various heart diseases. For example, its expression is upregulated in the right ventricular outflow tract of congenital heart disease patients [16]. Moreover, it suppresses cardiomyocyte proliferation. It has been found that miR-29a-3p could possess the protective function against cardiac fibrosis via inhibition of macrophage migration [17]. Also, upregulation of miR-29a-3p may protect cardiomyocytes from damage induced by hypoxia [18]. Our biochemical test results suggested that serum levels of TC, TG, and LDL-C were distinctly increased, and serum HDL-C levels were prominently decreased in the AS model. Nevertheless, miR-29a-3p mimic treatment improved serum levels of TC, TG, LDL-C, and HDL-C for the AS model. It has been acknowledged that TC, TG, and LDL-C are risk factors and HDL-C is a protective factor for AS [2]. More importantly, H&E and Masson's staining results confirmed that miR-29a-3p overexpression could prominently reduce arterial wall thickening as well as plaque formation of AS mice.

ox-LDL is a key risk factor for AS progression, which can affect the proliferation, migration, and invasion of VSMCs [19]. Thus, in this study, ox-LDL-induced VSMCs were utilized to construct an in vitro AS model. As expected, miR-29a-3p expression was prominently decreased with a concentration and time-dependent manner. Previous studies have shown that proliferative [20], migrated [21], and invaded [22] abilities of VSMCs are closely related to AS progression. After treatment with miR-29a-3p mimic, we found that the cell proliferative ability of VSMCs was suppressed according to CCK-8 and colony formation assays. Also, miR-29a-3p mimic transfection notably decreased the expression of Ki67 and PCNA induced by ox-LDL in VSMCs. Ki67 and PCNA are signs of cell proliferation and mediate DNA replication [23]. Our wound healing and Transwell assay results showed that miR-29a-3p mimic distinctly inhibited the migrated and invaded abilities of VSMCs induced by ox-LDL. Also, miR-29a-3p overexpression suppressed the expression of MMP9 and MMP2 proteins in VSMCs induced by ox-LDL. During the formation of atherosclerotic plaques, VSMCs migrate from the medium to the intima. VSMCs secrete MMPs, a family of zinc-dependent endopeptidases in the intima [24]. MMPs can promote the degradation of the extracellular matrix (ECM) as well as migration of VSMCs [25].

TNFRSF1A mRNA and protein expression was notably elevated both in the AS mouse model and ox-LDL-induced VSMCs, consistent with a previous study [26]. Intriguingly, after treatment with miR-29a-3p mimic, TNFRSF1A expression was remarkably inhibited. Emerging evidence suggests that miRNAs function via mediating the translation or stability of target mRNA [27–29]. As previous studies, TNFRSF1A could be mediated by microR-29c, thereby reducing poststroke depression [30]. Furthermore, TNFRSF1A regulated by miR-29a promotes AR42J cell apoptosis [31]. Our dual-luciferase report confirmed that TNFRSF1A could be targeted by miR-29a-3p. Further analysis found that TNFRSF1A overexpression could reverse the reduction of cell proliferative, migrated, and invaded abilities by miR-29a-3p overexpression in ox-LDL-induced VSMCs. Also, its upregulation distinctly inhibited the expression of Ki67, PCNA, MMP9, and MMP2 proteins, which were reversed by TNFRSF1A overexpression in ox-LDL-induced VSMCs.

However, several limitations of this study need to be pointed out. AS is a complex disease. Plaque progression involves different stages, from early atherosclerotic lesions to advanced lesions. During this process, the molecular players involved change continuously; the same can be true for potential target genes of miRNAs. Thus, further large-scale studies are needed to in-depth clarify the specific treatment mechanism of AS and transfer these findings to clinical research. Taken together, we conducted an in vivo model of AS by high-fat diet ApoE^−/−^ mice and an in vitro model by ox-LDL-exposed VSMCs. miR-29a-3p overexpression could improve plaque formation of AS and inhibit proliferation, migration, and invasion of ox-LDL-induced VSMCs via TNFRSF1A. The function of miR-29a-3p in AS is worthy of further analysis.

## 5. Conclusion

In this study, miR-29a-3p was downregulated and TNFRSF1A was upregulated both in the AS mouse model and ox-LDL-induced VSMCs. miR-29a-3p overexpression reduced plaque formation of AS mice. It could directly target the 3′UTR of TNFRSF1A. Moreover, miR-29a-3p overexpression suppressed cell proliferation, migration, and invasion of ox-LDL-induced VSMCs, which was reversed by TNFRSF1A overexpression. Thus, our findings could deepen the understanding about the molecular mechanisms of OS.

## Data Availability

The data used to support the findings of this study are available from the corresponding author upon request.

## Abbreviations

miRNAs: MicroRNAs
AS: Atherosclerosis
ox-LDL: Oxidized low-density lipoprotein
VSMCs: Vascular smooth muscle cells
H&E: Hematoxylin and eosin
3′UTR: 3′ untranslated region
TNFRSF1A: TNF receptor-1
TG: Triglyceride
LDL-C: Low-density lipoprotein cholesterol
HDL-C: High-density lipoprotein cholesterol
CCK-8: Cell counting kit-8.
